# Aging Injury Impairs Structural Properties and Cell Signaling in Human Red Blood Cells; Açaì Berry Is a Keystone

**DOI:** 10.3390/antiox12040848

**Published:** 2023-04-01

**Authors:** Sara Spinelli, Elisabetta Straface, Lucrezia Gambardella, Daniele Caruso, Giuseppe Falliti, Alessia Remigante, Angela Marino, Rossana Morabito

**Affiliations:** 1 Department of Chemical, Biological, Pharmaceutical and Environmental Sciences, University of Messina, 98122 Messina, Italy; saspinelli@unime.it (S.S.); rmorabito@unime.it (R.M.); 2 Biomarkers Unit, Center for Gender-Specific Medicine, Istituto Superiore di Sanità, 00161 Rome, Italylucrezia.gambardella@iss.it (L.G.); 3 Complex Operational Unit of Clinical Pathology of Papardo Hospital, 98166 Messina, Italy

**Keywords:** aging, oxidative stress, cytoskeleton proteins, band 3 tyrosine phosphorylation, Açaí berry extract, human red blood cells

## Abstract

Red blood cell (RBC) deformability is the ability of cells to modulate their shape to ensure transit through narrow capillaries of the microcirculation. A loss of deformability can occur in several pathological conditions, during natural RBC aging through an increase in membrane protein phosphorylation, and/or through the structural rearrangements of cytoskeletal proteins due to oxidative conditions, with a key role played by band 3. Due to the close relationship between aging and oxidative stress, flavonoid-rich foods are good candidates to counteract age-related alterations. This study aims to verify the beneficial role of Açaì extract in a d-Galactose (d-Gal)-induced model of aging in human RBCs. To this end, band 3 phosphorylation and structural rearrangements in membrane cytoskeleton-associated proteins, namely spectrin, ankyrin, and/or protein 4.1, are analyzed in RBCs treated with 100 mM d-Gal for 24 h, with or without pre-incubation with 10 μg/mL Açaì extract for 1 h. Furthermore, RBC deformability is also measured. Tyrosine phosphorylation of band 3, membrane cytoskeleton-associated proteins, and RBC deformability (elongation index) are analyzed using western blotting analysis, FACScan flow cytometry, and ektacytometry, respectively. The present data show that: (i) Açaì berry extract restores the increase in band 3 tyrosine phosphorylation and Syk kinase levels after exposure to 100 mM d-Gal treatment; and (ii) Açaì berry extract partially restores alterations in the distribution of spectrin, ankyrin, and protein 4.1. Interestingly, the significant decrease in membrane RBC deformability associated with d-Gal treatment is alleviated by pre-treatment with Açaì extract. These findings further contribute to clarify mechanisms of natural aging in human RBCs, and propose flavonoid substances as potential natural antioxidants for the treatment and/or prevention of oxidative-stress-related disease risk.

## 1. Introduction

The aging process is characterized by a gradual accumulation of damage to cells, progressive functional decline, and increased susceptibility to chronic disease [1]. A causal hypothesis that has gained considerable interest in recent years postulates that pathophysiological changes during aging are due to progressive oxidative damage to cellular macromolecules [2,3,4,5,6]. During cellular metabolism, the production of reactive oxygen and/or nitrogen species (ROS and/or RNS) generated in biological systems is balanced by the ability of the latter to protect themselves through their endogenous antioxidant machinery [7,8,9,10]. Nevertheless, when oxidants are generated in excess, or when the endogenous antioxidant defenses are inefficient, this balance could be perturbed, thus resulting in oxidative stress [11,12,13,14,15]. In these conditions, macromolecular targets (lipids, proteins, and nucleic acids) can be altered through oxidation to an extent that exceeds repair capacity [16,17,18]. Although several cell models have been employed to explore the biophysical and/or biochemical changes during aging, red blood cells (RBCs) have superiority amongst them [3]. The red blood cell’s functional role is oxygen transport from the lungs to the tissues—and vice versa—providing all cells with the required oxygen. To fulfill their tasks, RBCs are capable of elastically deforming during mechanical stress and pass via the narrow microcirculation capillaries [19,20]. There is compelling evidence that treatment of RBCs with oxidizing compounds not only impair their deformability but also change their shape, thus suggesting a key role of intracellular redox status, both in structural characteristics and the control of deformability [21,22,23,24,25,26]. Surprisingly, a close relationship between oxidative stress and RBC deformability has also been reported in experimental investigations involving patients with sickle cell disease. In disease states, ROS-generated injury to the membrane components of RBCs is thought to favor membrane fragility and rigidity, thus resulting in hemoglobin release into the plasma (intravascular hemolysis), as well as systemic NO (nitric oxide) scavenging [22]. Despite the bioclinical significance of these events, the basic biochemistry of the processes controlling normal RBC deformability in both health and disease as well as the underlying signaling pathways still are poorly clarified [27].

**Figure 1:**
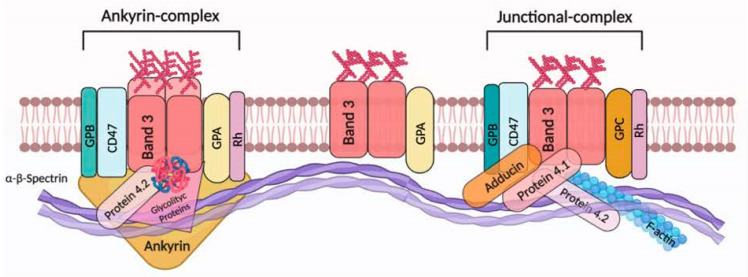
Band 3 interactions in the human RBC membrane. In RBCs, band 3 presents as an oligomeric form (dimers and/or tetramers) or alternatively as larger aggregates [35]. Dimeric band 3 protein complex is composed of freely moving band 3 dimers with glycophorin A (GPA). However, band 3 molecules (as dimers) are thought to be associated with junctional complexes. In particular, this multiprotein complex is represented by band 3 dimers, adducin, protein 4.1, glycophorin C (GPC) and Rh. Meanwhile, the band 3 tetrameric complex (the ankyrin complex) is organized as a tetramer of band 3 bound to protein 4.2 and ankyrin, alongside GPA. Ankyrin/protein 4.1 and/or protein 4.2/actin binding to band 3 facilitates the association with the underlying α-β-spectrin cytoskeleton [30]. Sedimentation and extractability experiments indicate that both glycolytic proteins and deoxyhemoglobin are associated with the tetrameric form of band 3. Recently, it was demonstrated that Rh protein, CD47, and glycophorin B (GPB) are associated with band 3 (tetrameric form) [35]. This figure was created using BioRender.com.

The biophysical properties and distribution of the cytoskeletal proteins are responsible for their normal biconcave shape. The structure of the RBC membrane cytoskeleton is composed of a protein network of mainly spectrin, which maintains RBC geometry by changing shape under the mechanical stress influence [28,29]. The network is characterized by the interaction between spectrin (two heterodimers: α- and β-subunits) anchored to the RBC membrane at two points: the ankyrin complex and the junctional complex, both of which center in band 3 (Figure 1) [30]. Protein–protein interactions are modulated by post-translational modifications, mainly by phosphorylation of tyrosine and/or serine protein residues, leading to conformational changes of protein molecular structure [29,31]. Protein dephosphorylation and phosphorylation play a crucial role to regulate elasticity of the plasma membrane and, in turn, RBC deformability [32,33,34]. One of the major objectives of RBC redox regulation results in the principal membrane-spanning protein: band 3 [35]. Band 3 promotes anion exchange (HCO_3_^−^ and Cl^−^) across the plasma membrane of RBCs [36], anchors the α-β-spectrin cytoskeleton to the lipid bilayer [37], promotes and regulates glycolytic enzymes [38], participates in the control of RBC lifespan [39], and serves as a docking site for different membrane proteins including ankyrin and protein 4.1 [40]. In addition, band 3 is a prominent substrate of serine/threonine kinases [34,41]. In fact, it has been widely reported that oxidized band 3 is phosphorylated. Specifically, it was demonstrated with mass spectrometry that the primary band 3 phosphorylation appears at tyrosine 8 and 21 by Syk kinase, while the secondary phosphorylation by Lyn kinase occurs at tyrosine 359 and 904 level [42].

In the blood flow, RBCs are constantly exposed to reactive oxygen and/or nitrogen species (ROS and/or RNS), possibly damaging RBCs and thus impairing their functions. To reduce the ROS effect and the related oxidative stress [21], RBCs are equipped with good antioxidant machinery involving both non-enzymatic antioxidants such as ascorbic acid and glutathione, and enzymatic antioxidants including catalase, superoxide dismutase, peroxiredoxin-2, and glutathione peroxidase, in order to provide antioxidant protection not only to themselves, but also to other body tissues and organs [28]. Besides the intracellular mechanisms, much consideration has been addressed at the antioxidant role of some molecules supplied by the diet [43]. *Euterpe oleracea* (known as Açaì) is a typical Amazonian Brazilian fruit [44,45,46,47] that displays different bioactive compounds with several properties, for example antioxidant activities. Açaì’s biological effects are due to its chemical composition, which includes several phytochemical compounds such as flavonoids [48], which are able to neutralize ROS and inactivate pro-oxidant molecules [49,50]. In a previous study [51], we demonstrated that freeze-dried Açaì extract exerts beneficial effects against some processes related to accelerated aging induced using d-Galactose (d-Gal) treatment in human RBCs. Specifically, Açaì extract prevented leptocyte formation observed after d-Gal treatment as well as d-Gal-induced oxidative stress injury (namely ROS production, lipid-peroxidation, and as protein sulfhydryl group oxidation), while restoring alterations in band 3 and CD47 distribution. Interestingly, d-Gal exposure was also associated with hemoglobin formation and the acceleration of the rate constant of SO_4_^2−^ uptake via band 3. Both alterations were attenuated with Açaì extract pre-treatment [51]. 

Based on the obtained data, the aim of this study was to verify the beneficial role of Açaì extract, both on band 3 phosphorylation, and structural rearrangements in membrane cytoskeleton-associated proteins, namely α-β-spectrin, ankyrin, and/or protein 4.1, in RBCs treated with 100 mM d-Galactose (d-Gal). Both events (band 3 phosphorylation and/or rearrangements in cytoskeleton proteins) might contribute to cellular deformation and structural instability of human RBCs. Thus, deformability of RBCs was also determined using the elongation index in relation to the shear stress applied to the RBC membrane.

## 2. Materials and Methods

### 2.1. Material and Stock Solutions

Chemical substances were purchased from Sigma Aldrich. All homemade solutions were prepared using Milli-Q-quality water. Working solutions of d-Gal were prepared by diluting 1 M stock in isotonic solution (150 mM NaCl and 5 mM HEPES, pH 7.4, 300 mOsm/kg H_2_O). Açaì extract was resuspended in distilled water. The substance (Cas Number: 879496-95, 906351-38-0) was purchased from Farmalabor Srl (Canosa di Puglia, Barletta, Italy).

### 2.2. Preparation of RBC Samples

This investigation involving human participants was approved by a local Ethics Committee (prot.52-22, 20 April 2022) in accordance with the Helsinki Declaration. Whole human blood from healthy volunteers (20–60 years old) was, upon informed consent, placed in test tubes with EDTA. Red blood cells were washed in isotonic solution (150 mM NaCl and 5 mM HEPES, 5 mM Glucose, pH 7.4, 300 mOsm/kg H_2_O) and centrifuged thrice (Neya 16R, 1200× *g*, 5 min) to discard both buffy coat and plasma. Then, RBCs were suspended at definite hematocrits in isotonic solution and subjected to different analyses.

### 2.3. Analytical Cytology

Red blood cells, either untreated or incubated with isotonic solutions containing D-Gal, and either with or without pre-exposure to Açaì extract, were fixed with 3.7% formaldehyde in PBS (pH 7.4) for 10 min at 25 °C and then washed in the same PBS buffer. Then, cells were permeabilized with 0.5% Triton X-100 in PBS for 5 min at 25 °C. Blood cells were first incubated with monoclonal anti-(α-β)-spectrin (Sigma, Burlington, MA, USA), monoclonal anti-ankyrin (Invitrogen Thermo Fisher, Waltham, MA, USA), or monoclonal anti-protein 4.1 (Santa Cruz Biotechnology, Dallas, TX, USA) antibodies for 30 min at 37 °C, and successively incubated with a fluorescein isothiocyanate (FITC)-labeled anti-mouse antibody (Sigma) for 30 min at 37 °C [52]. Red blood cells were exposed to the secondary antibody. Samples were processed with an Olympus BX51 fluorescence microscope or a FACScan flow cytometer (Becton Dickinson, Franklin Lakes, NJ, USA) supplied with an argon laser of 488 nm. The average values of fluorescence intensity were represented in a quantitative analysis. The values of fluorescence intensity were normalized for those untreated RBCs and are shown in percentages (%).

### 2.4. Preparation of RBC Membranes

Cell membranes were processed as described by other researchers [53], with small changes. Red blood cells were suspended in cold solution (1.5 mL of 2.5 mM NaH_2_PO_4_) containing an inhibitor mixture. Blood samples were centrifuged (Eppendorf, 4 °C, 18,000× *g*, 10 min) to discard hemoglobin. Membranes were then solubilized with SDS (1% *v*/*v*) and put on ice for 20 min. The supernatant containing the solubilized membrane proteins was conserved at −80 °C.

#### SDS-PAGE Preparation and Western Blotting Analysis

Red blood cell membranes were heated for 10 min at 95 °C after dissolving in Laemmli buffer [54]. The proteins were separated using SDS-polyacrylamide gel electrophoresis and transferred to a polyvinylidene fluoride membrane by maintaining a constant voltage for 2 h. Membranes were blocked for 1 h at 25 °C in BSA and incubated at 4 °C with the primary antibodies: monoclonal anti-p-TyR (tyrosine) antibody (T1325, Sigma-Aldrich, Milan, Italy) produced in mice and diluted 1:1000 in TBST; and monoclonal anti-Syk antibody (SAB4500552, Sigma-Aldrich, Milan, Italy) created in rabbit and diluted 1:500 in TBST). Successively, membranes were incubated for 1 h with peroxidase-conjugated goat anti-mouse/rabbit IgG secondary antibodies (A9044/A0545, Sigma-Aldrich, Milan, Italy) diluted 1:10000/1:20000 in TBST solution at 25 °C. To quantify the protein in equal amounts, a monoclonal anti-β-actin antibody (A1978, Sigma-Aldrich, Milan, Italy), diluted 1:10000 in TBST solution and created in mice, was incubated with the same membrane, as indicated by Yeung and co-authors [55]. A system of chemiluminescence detection (Super Signal West Pico Chemiluminescent Substrate, Pierce Thermo Scientific, Rockford, IL, USA) was employed to obtain the signal, whose images were transferred to a software for analysis (Image Quant TL, v2003, Sunnyvale, CA, USA). The intensity of protein bands was determined with densitometry (Bio-Rad ChemiDocTM XRS+, Hercules, CA, USA).

### 2.5. Red Blood Cell Deformability Measurement

The deformability of RBCs was determined using ektacytometry (LORRCA; Mechatronics Instruments BV, AN Zwaag, Netherlands). We used an analytical method suggested by Donadello and collaborators [56]. The elongation index (EI) was obtained with the following equation: EI = (L − W)/(L + W); where L and W indicate the length and width of the diffraction pattern. A major RBC deformability corresponds to a higher EI for all shear stresses. We evaluated the EI curves for 12 values of shear stress, since RBC deformability achieves a plateau at 50 Pa. From these curves of shape modifications, the maximal elongation (EI max) was calculated. The curves are represented in logarithmic scales.

### 2.6. Experimental Data and Statistics

All obtained data are provided as arithmetical means ± SEM. We used GraphPad Prism (version 9.0) for statistical analysis and Excel (version 2019) software for graphics. Significant differences of the values were analyzed with the one-way ANOVA, followed by Bonferroni’s multiple comparison post-test. Significant differences were assumed at *p* < 0.05; (n) corresponds to the independent measurements.

## 3. Results

### 3.1. Detection of Band 3 Tyrosine Phosphorylation and Syk Kinase Levels

**Figure 2:**
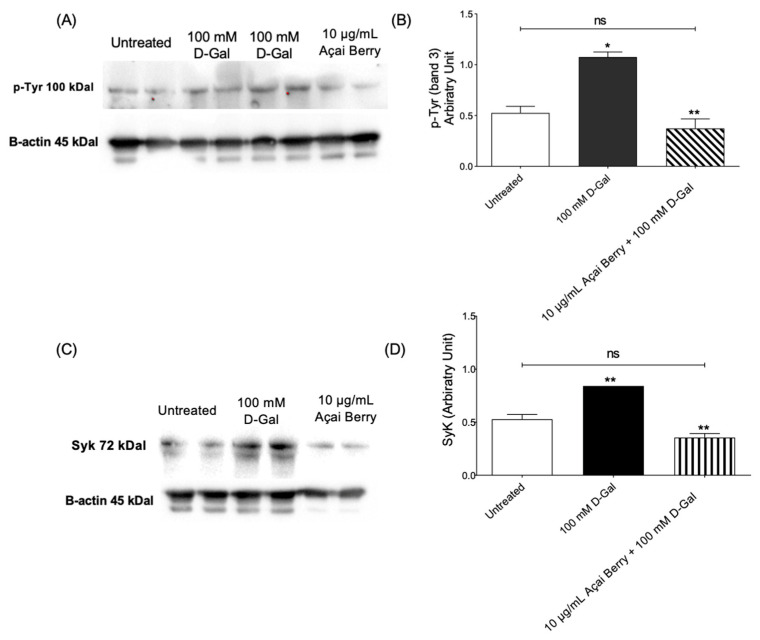
Measurement of protein levels using western blotting analysis. (**A**,**B**) p-Tyr (tyrosine) and (**C**,**D**) Syk levels determined in the control or exposed to 100 mM D-Gal for 24 h with or without pre-exposure to 10 µg/mL Açai Berry extract for 1 h. ns—not significant versus left untreated; * *p* < 0.05 and ** *p* < 0.01 versus left untreated (control); n = 3.

Figure 2 shows the tyrosine phosphorylation levels of band 3 as well as Syk kinase levels in RBCs incubated with 100 mM d-Gal (24 h at 25 °C) with or without pre-treatment (10 µg/mL Açaì extract for 1 h). Exposure to 100 mM d-Gal caused an intense phosphorylation of band 3 at the level of tyrosine 8 and tyrosine 21, respectively (Figure 2A,B). Importantly, pre-treatment with freeze-dried Açaì extract prevented the increase in tyrosine phosphorylation of band 3 in 100 mM d-Gal-treated RBCs (Figure 2A,B). Freeze-dried Açaì extract alone did not significantly impair tyrosine phosphorylation levels. In parallel, Syk kinase, responsible for the phosphorylation of tyrosine 8 and 21 of band 3, was detected (Figure 2C,D). In RBCs pre-incubated with 10 µg/mL Açaì extract and then treated with 100 mM d-Gal, Syk kinase expression was reduced compared to those measured in 100 mM d-Gal-treated RBCs. It is noteworthy that Açaì extract did not significantly impact Syk kinase levels (results not shown).

### 3.2. Detection of α- and β-Spectrin Levels

**Figure 3:**
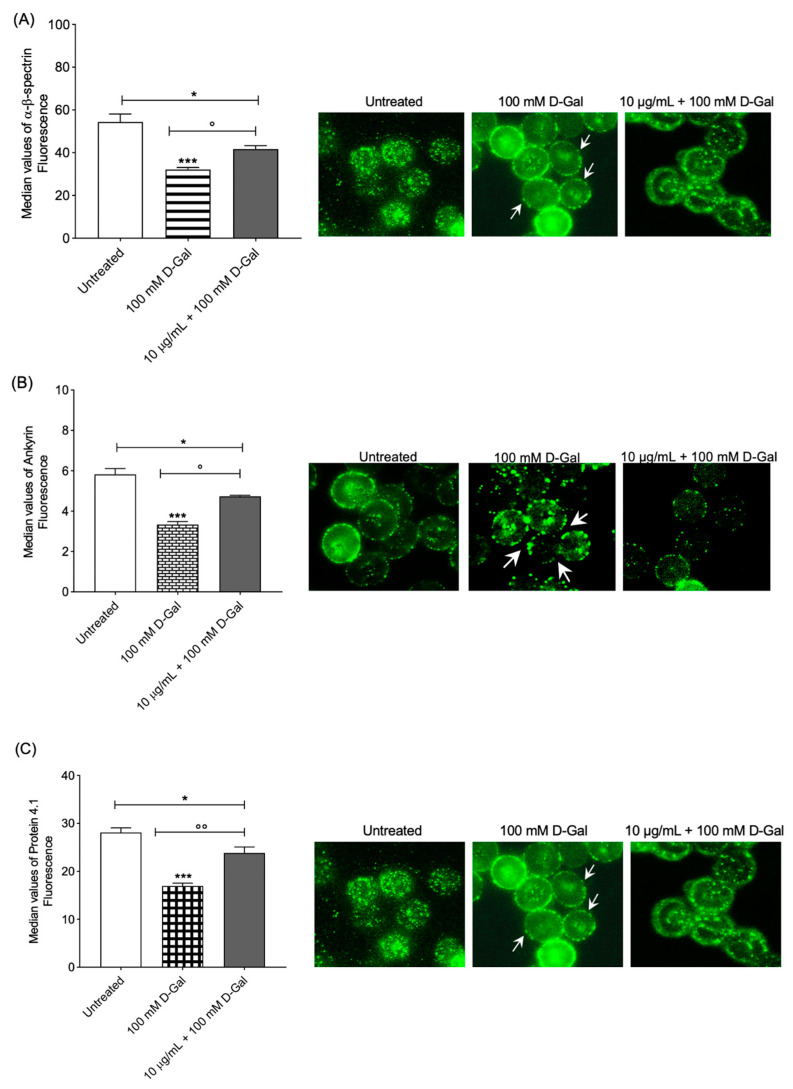
(**A**) Analysis using flow and static cytometry of α- and β-spectrin expression. Red blood cells were treated for 24 h with 100 mM d-Gal, with or without pre-incubation for 1 h with 10 µg/mL Açaì extract. Bars represent fluorescence intensity values. In panel (**A**) (right), representative images of α- and β-spectrin levels obtained using immunofluorescence are reported. Samples were observed with a 100× objective. Note the morphological changes in 100 mM d-Gal (arrows). * *p* < 0.05 control; *** = *p* < 0.001 control; ° = *p* < 0.05 versus 100 mM d-Gal; n = 4. (**B**) Analysis using flow and static cytometry of ankyrin expression. Red blood cells were treated for 24 h with 100 mM d-Gal, with or without pre-incubation for 1 h with 10 µg/mL Açaì extract. Bars represent fluorescence intensity values. In panel (**B**) (right), representative images of ankyrin levels obtained using flow cytometry immunofluorescence are reported. Cell samples were observed with a 100× objective. Note the morphological changes in 100 mM d-Gal (arrows). * = *p* < 0.05 left untreated (control); *** = *p* < 0.001 control; ° = *p* < 0.05 versus 100 mM d-Gal; n = 4. (**C**) Analysis using flow and static cytometry of protein 4.1 expression. Red blood cells were treated for 24 h with 100 mM d-Gal, with or without pre-incubation for 1 h with 10 µg/mL Açaì extract. Bars represent fluorescence intensity values. In panel (**C**) (right), representative images of protein 4.1 levels obtained using flow cytometry immunofluorescence are reported. Cell samples were observed with a 100× objective. Note the morphological changes in 100 mM d-Gal (arrows). * = *p* < 0.05 left untreated; *** = *p* < 0.001 left untreated; °° = *p* < 0.01 versus 100 mM d-Gal; n = 4.

The content and localization of α- and β-spectrin were evaluated in RBCs treated with 100 mM d-Gal for 24 h compared to controls (Figure 3A) with flow cytometry analysis. In RBCs pre-incubated with 10 µg/mL Açaì extract and then treated with 100 mM d-Gal, α- and β-spectrin expression levels were significantly higher than those of RBCs treated with 100 mM d-Gal, and were significantly different with respect to the control (Figure 3A, left panel). However, the expression of these proteins was not completely recovered using pre-incubation with 10 µg/mL Açaì extract. Freeze-dried Açaì extract alone did not significantly affect α- and β-spectrin expression levels (results not shown). In Figure 3A (right panel), three representative immunofluorescence images are shown. An intense rearrangement and redistribution (arrows) of α- and β-spectrin was detected especially after treatment with 100 mM d-Gal alone.

### 3.3. Detection of Ankyrin Expression Levels

Regarding ankyrin, flow cytometry analysis showed decreased expression levels in RBCs exposed to 100 mM d-Gal for 24 h compared to controls (Figure 3B). In RBCs pre-incubated with 10 µg/mL of Açaì extract and then treated with 100 mM d-Gal, ankyrin expression was higher than those of RBCs treated with 100 mM d-Gal and significantly different with respect to the control (Figure 3B, left panel). Moreover, the protein expression was not completely recovered by treatment with 10 µg/mL Açaì extract. Açaì extract did not impact ankyrin levels (results not shown). Data acquired using flow cytometry were validated with immunofluorescence analyses, which reported an intense redistribution and rearrangement (arrows) of ankyrin (Figure 3B, right panel).

### 3.4. Detection of Protein 4.1 Levels

Protein 4.1 levels were decreased in human RBCs incubated with 100 mM d-Gal for 24 h with respect to those controls (Figure 3C). In RBCs pre-incubated with 10 µg/mL of Açaì extract and then treated with 100 mM d-Gal, protein 4.1 levels were higher than those of RBCs treated with 100 mM d-Gal, but different with respect to the controls (Figure 3C, left panel). The levels of this protein were not completely restored with 10 µg/mL Açaì pre-treatment. Açaì extract did not influence protein 4.1 expression levels (results not shown). In addition, protein 4.1 redistribution was detected with immunofluorescence (Figure 3C, right panel). In particular, protein 4.1 was mainly clustered in leptocytes following the treatment with 100 mM d-Gal with respect to control RBCs. These alterations were partially thinned using Açaì extract pre-treatment.

### 3.5. Human RBC Deformability Measurement

**Figure 4:**
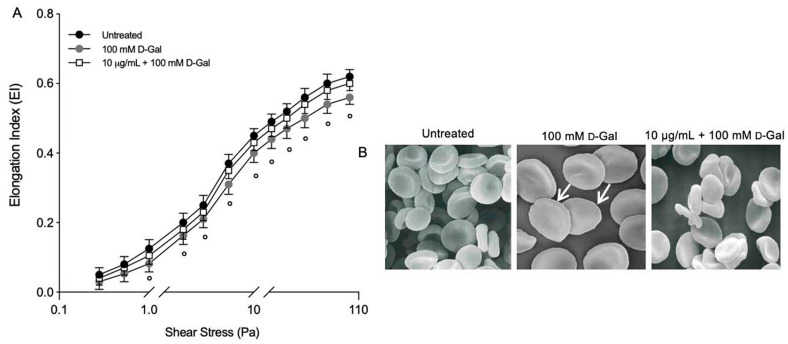
(**A**) Elongation index (EI) at different shear stress values (Pa). Red blood cells were exposed to 100 mM d-Gal for 24 h, with or without pre-incubation with 10 µg/mL Açaì extract for 1 h. ° = *p* < 0.05 versus 100 mM d-Gal; n = 3. (**B**) Evaluation of RBC morphology. Representative images obtained using scanning electron microscopy show RBCs with normal biconcave form (control) and with flattened shape (leptocytes, arrows) (100 mM d-Gal). Açaì extract (10 µg/mL) pre-incubation attenuated the shape changes compared to 100 mM d-Gal treatment. Magnification 3000×. Images reported from reference cited above.

For most shear stresses investigated, deformability (EIs) in RBCs treated with 100 mM d-Gal for 24 h was significantly lower than left untreated RBCs (Figure 4A). The EI max for d-Gal-treated samples was 0.56 compared to 0.62 for controls. In RBCs pre-incubated with 10 µg/mL of Açaì extract and then incubated with 100 mM d-Gal, the EI max was higher than that of RBCs treated with 100 mM d-Gal, but was no different with respect to the untreated RBCs. The EI max for freeze-dried Açaì extract-treated samples was 0.60. A decrease in deformability resulted in altered cell shape. Thus, RBC morphology alteration is also reported. These data were obtained in a previous study [51] but now support the loss of deformability in RBC-treated with D-Gal (Figure 4B). Using scanning electron microscopy, we detected 28.2% of leptocytes in RBCs treated with 100 mM d-Gal for 24 h. However, 10 μg/mL Açaì extract pre-treatment induced a reduction of the percentage of structurally altered RBCs. In particular, the percentage of leptocytes was reduced to 12% following pre-treatment with freeze-dried Açaì extract.

## 4. Discussion

Red blood cells flow through circulatory system for 120 days, during which they continuously modify their biconcave shape (8 µm size) to able to traverse blood vessels that narrow to 1 µm size. Deformability of RBCs is, then, an essential biomechanical property to maintain proper blood flow in the microcirculation, in order to allow cells to play out their essential functions [29]. Changes to membrane and/or cytoskeleton proteins, including post-translational modifications, are probably responsible for the impaired cell deformability associated with oxidative stress and natural aging processes [21,22,57,58,59]. Studying the molecular deformability is of primary importance to understanding RBC physiology and/or pathophysiology. Increasingly, researchers have focused on natural antioxidants and their capacity to neutralize oxidative stress-induced pathological conditions. Indeed, phytochemical-rich dietary supplementation was found to provide several health benefits [25,43,44] and, in this regard, Açai berry represents a good candidate showing different beneficial activities, such as antioxidant properties in various experimental models (in vitro and ex vivo) [60,61,62].

Herein, we investigated the protective role of Açai berry both on tyrosine phosphorylation of band 3 and the structural rearrangements in membrane cytoskeleton-associated proteins (α- and β-spectrin, protein 4.1, and ankyrin) in a model of aging induced using d-Gal exposure in human RBCs. The present findings contribute to further elucidate the mechanisms of aging in human RBCs and indicate that the phytochemicals in the Açaì fruit could improve the structural/functional changes of human RBCs, thus possibly preventing pathological events attributable to oxidative stress-related aging.

It is well documented that RBCs counteract oxidation with metabolic feedback addressed to improve the production of NADPH and restore GSH and thioredoxin levels. Pari passu, RBCs also activate tyrosine kinases able to induce phosphorylation of band 3 tyrosine, the most represented RBC membrane protein as well as the most significant linkage between the lipid bilayer and the cytoskeleton [36]. In RBCs, band 3 hyperphosphorylation was demonstrated in both malaria and pro-oxidant hemolytic disorders, but the underlying mechanisms leading to its phosphorylation and pathophysiological significance have been only partially characterized [34,63,64,65]. The redox regulation of band 3 tyrosine phosphorylation involves two different enzymes: Lyn is responsible for the phosphorylation of 359 tyrosine, while Syk is responsible for the phosphorylation of 8 and 21 tyrosine [42,64]. It was proposed that phosphorylation affects cytoskeleton (spectrin)band 3 binding and provokes changes in the deformability and shape of human RBCs [66]. In this context, d-Gal-induced oxidative stress caused an intense tyrosine phosphorylation of band 3; however, the pre-treatment with 10 µg/mL freeze-dried Açaì totally restored the post-translational modification (Figure 2A–C). Since Syk kinase was reported to be primarily responsible for tyrosine 8 and 21 phosphorylation in the intracellular domain of oxidized band 3 [67], to better explore the role of Syk kinase, its enzyme expression was also evaluated. In particular, these data showed a net increase in the Syk kinase content in RBCs treated with 100 mM d-Gal, thus confirming that Syk kinase could act mainly on oxidized band 3 [53]. Vice versa, Syk basal levels were restored with pre-treatment with 10 µg/mL Açaí extract (Figure 2B–D). Band 3 cytoplasmatic domain oxidation, probably caused by an increase in intracellular ROS production, was able to activate the Syk docking to band 3 and inhibit tyrosine phosphatases [53,68]. In addition, band 3 hyper-tyrosine phosphorylation provoked relevant alterations in RBC features, such as the disruption of the band 3-ankyrin bound that connects spectrin to the plasma membrane, as well as metabolic alterations via anion transport alterations in band 3. With regard to this latter point, band 3 anion exchange capability [69,70,71,72,73,74,75,76,77] represents a suitable method to explore functional features by determining the rate constant for SO_4_^2−^ uptake [78]. The evidence that band 3 displays alterations in the rate constant for SO_4_^2−^ uptake after the treatment of RBCs to d-Gal-induced oxidation has also been previously showed. Specifically, d-Gal-caused oxidative stress induced an acceleration of the anion exchange; however, pre-treatment with Açaí extract completely restored the rate constant of SO_4_^2−^ uptake, thus demonstrating the beneficial effect of the Açaí extract on band 3 anion exchange function [51]. Such findings prompted us to go deeper into the mechanisms underlying the d-Gal effects on band 3, leading us to consider the biomechanical implications of band 3 alterations, possibly prevented by Açaí extract.

As mentioned above, damage to membrane and/or cytoskeletal proteins generated by oxidative stress contribute to the impairment of RBC deformability [79]. The RBC cytoskeleton structure is an internet structure organized by proteins, mainly α- and β-spectrin, that contribute to the RBC’s physiological structure by changing under the influence of mechanical stress [80,81,82,83]. The network of α- and β-spectrin binds to the lipid membrane matrix via integral proteins, including band 3 [35]. In response to oxidative stress, band 3 phosphorylation on tyrosine residues greatly increases, thus leading to structural rearrangements of the α- and β-spectrin [84,85,86]. In this regard, treatment with d-Gal caused both redistribution and loss of α- and β-spectrin expression on the RBC membrane (Figure 3C). The latter result was supported by the presence of cells with an atypical shape (seen in scanning electron microscopy images), namely leptocytes (Figure 4B). In this aging model, spectrin levels and its distribution were not completely recovered in RBCs pre-treated with 10 µg/mL of Açaí extract. This result is also confirmed by a partial restoration of RBC morphological characteristics in scanning electron microscopy images (Figure 4B).

The cytoskeleton structure is anchored to the RBC plasma membrane at two points. The first one is the network assembly of α- and β-spectrin, protein 4.1, and actin (junctional complex); the second one is attached to the membrane network by the band 3/ankyrin protein complex (Figure 1). Plasma membrane and cytoskeletal protein interactions determine the membrane stability and modulate the deformability of RBCs under shear stress [30,37,87]. Regarding protein 4.1, flow cytometry analysis revealed a significant decrease in expression in RBCs treated with d-Gal compared to untreated cells (Figure 2B). Açaì extract pre-treatment partially restored protein 4.1 expression levels in d-Gal-treated RBCs. Additionally, ankyrin expression was also found to be significantly decreased in human RBCs treated with 100 mM d-Gal with respect to those untreated (Figure 2B). This evidence is supported by the images obtained using flow cytometry immunofluorescence. Unfortunately, freeze-dried Açaì extract pre-treatment did not totally restore ankyrin levels in RBCs treated with 100 mM d-Gal for 24 h (Figure 2B). The deficiency of ankyrin results in incisive changes in RBC morphology and a shortened RBC lifespan, whereas the absence of protein 4.1 causes only a slight modification in RBC morphology and survival, thus suggesting that the glycophorin C and/or protein 4.1 linkage is functionally less significant than the band 3-ankyrin-spectrin linkage [88,89]. In oxidative stress conditions, the activation of caspase-3 can cleave the cytoplasmic end of band 3, impacting the interactions of band 3 with cytosolic proteins and the binding to ankyrin and the cytoskeleton, thus inducing the phosphatidylserine exposure [90].

As mentioned above, protein–protein interactions are modulated by post-translational modifications, mostly by phosphorylation of protein residues which leads to conformational modifications of protein structure [29]. These data confirm that the phosphorylation of membrane and/or cytoskeletal proteins (e.g., band 3 by Syk kinase) damages their affinity for interacting partners and favors the disassociation of the α- and β-spectrin network, which may create a more flexible or relaxed cytoskeletal structure and a reduction in RBC membrane stability. Moreover, band 3 hyper-phosphorylation was also linked to glucose-6-phosphate dehydrogenase absence and senescence of RBCs; all pathological conditions characterized by an increase in RBC oxidative stress and band 3 clustering [34,63,91]. To date, genetic defects in α- and β-spectrin, or in proteins that engage the α- and β-spectrin network to the lipid bilayer (e.g., band 3, ankyrin, and/or protein 4.1), induce a loss of deformability and elasticity. The increase in RBC fragility and cell fragmentation in mice and humans could produce abnormally shaped RBCs that are rapidly removed by the spleen [92,93,94,95].

Circulating cell morphology has a fundamental influence on the blood rheological properties, and changes in morphology can lead to increased aggregation and decreased deformability [96,97]. It is well established that d-Gal induces both protein damage and lipid peroxidation in RBCs, either of which can be responsible for impacting deformability adversely [51]. Using flow cytometry and ektacytometry, we observed a marked change in RBC membrane deformability and shape in d-Gal-treated samples, and we reported that these changes are associated with a total restoration after 10 µg/mL pre-treatment of freeze-dried Açaì extract (Figure 4A). The decreased deformability in RBCs is also observed in pathological conditions linked to decreased NO bioavailability, such as hypercholesterolemia, diabetes mellitus, and hypertension. The NO treatment was shown to affect RBC deformability, thus implying that the ROS balance impacting the RBC membrane and cytoskeletal proteins is strictly related to RBC deformability. In this context, melatonin prevented lipid peroxidation in RBCs and enhanced deformability in samples exposed to oxidative stress induced with NO incubation [82].

The plasma membrane is important not only for cellular integrity but also for cellular function. For example, protein oxidation injury can modify RBC deformability by affecting ion exchange mechanisms [98,99]. In particular, free radicals dwindle the band 3 function, resulting in increased anion transport across the membrane, a reduction in cell stability, and subsequently a reduction in deformability [100]. Moreover, peroxyl radicals also inhibit the RBC Ca^2+^-Mg^2+^ ATPase activity, with an increase in the intracellular calcium causing an impairment in RBC deformability. Elevation of oxidative stress and cytosolic Ca^2+^ increases could activate RBC scramblase and calpain, resulting in phosphatidylserine externalization and RBC membrane blebbing, respectively [62,63]. The phosphatidylserine externalization provokes RBC phagocytosis and then their elimination from blood flow. However, no externalization of phosphatidylserine was reported in RBCs exposed to d-Gal, thus denoting that RBCs do not perform eryptosis but stay in an early phase of the oxidative stress process, which is very important to promote the action of antioxidant molecules. In our aging model, where phosphatidylserine scrambling did not occur, derangements related to oxidation increase could be reversed using Açai berry extract treatment, which is particularly rich in flavonoids [64]. A similar condition could also be detected in oxidative stress-related diseases, including cardiovascular complications, anemia, diabetes, and chronic kidney disease [101,102]. Hence, the protective effects of flavonoids (Açai berry) apply to an early natural aging state.

## 5. Conclusions

The plasma membrane of RBCs is organized in a bilayer and an underlying cytoskeleton of proteins; the physical and mechanical characteristics of this structure are the fundamental determinants of cellular shape and the response of the cells to exogenous stressors, including aging-related oxidative stress. In light of the results obtained here, we demonstrated that: (i) Açaì berry extract pre-treatment restores both the increase in band 3 tyrosine phosphorylation and Syk kinase levels in RBCs exposed to 100 mM d-Gal treatment; (ii) Açaì berry extract pre-treatment partially restores the alterations in the distribution of membrane cytoskeleton-associated proteins, namely α- and β-spectrin, ankyrin, and protein 4.1; and (iii) Açaì berry extract pre-treatment attenuates the significant decrease in RBC membrane deformability associated with 100 mM d-Gal treatment. These findings reveal that oxidative stress-related alterations might be reversible, and that early application of antioxidant molecules might be helpful to counteract oxidative stress-induced derangements. In addition, the present study further provides mechanistic insights into the benefits arising from the use of natural flavonoids against aging-related oxidative stress on a cellular level.

## Data Availability

Data is contained within the article.
